# Network-based inference of master regulators in epithelial membrane protein 2-treated human RPE cells

**DOI:** 10.1186/s12863-022-01047-9

**Published:** 2022-07-07

**Authors:** Hua Wan, Wei Gao, Wei Zhang, Zijiao Tao, Xiang Lu, Feng Chen, Jian Qin

**Affiliations:** 1 Sir Run Run Hospital, Nanjing Medical University, 109 Longmian Road, Nanjing, 211100 China; 2 Department of Healthcare management, Sir Run Run Hospital, Nanjing Medical University, 109 Longmian Road, Nanjing, 211100 China; 3 Nanjing Medical University, 101 Longmian Road, Nanjing, 211166 China

**Keywords:** Differentially expressed genes (DEGs), Epithelial membrane protein 2 (*EMP2*), Retinal pigment epithelial cells (*hRPECs*), Computational inference, Gene expression, Master regulators (MRs), Transcription regulatory networks (TRNs), Systems biology

## Abstract

**Background:**

The application of cell-specific construction of transcription regulatory networks (TRNs) to identify their master regulators (MRs) in *EMP2* induced vascular proliferation disorders has been largely unexplored.

**Methods:**

Different expression gene (DEGs) analyses was processed with DESeq2 R package, for public RNA-seq transcriptome data of *EMP2*-treated hRPECs versus vector control (VC) or wild type (WT) hRPECs. Virtual Inference of protein activity by Enriched Regulon analysis (VIPER) was used for inferring regulator activity and ARACNE algorithm was conducted to construct TRNs and identify some MRs with DEGs from comparisons.

**Results:**

Functional analysis of DEGs and the module analysis of TRNs demonstrated that over-expressed *EMP2* leads to a significant induction in the activity of regulators next to transcription factors and other genes implicated in vasculature development, cell proliferation, and protein kinase B signaling, whereas regulators near several genes of platelet activation vascular proliferation were repressed. Among these, *PDGFA, ALDH1L2, BA1AP3, ANGPT1 and ST3GAL5* were found differentially expressed and significantly activitve in *EMP2*-over-expressed *hRPECs* versus vector control under hypoxia and may thus identified as MRs for *EMP2*-induced lesion under hypoxia.

**Conclusions:**

MRs obtained in this study might serve as potential biomarkers for *EMP2* induced lesion under hypoxia, illustrating gene expression landscapes which might be specific for diabetic retinopathy and might provide improved understanding of the disease.

**Supplementary Information:**

The online version contains supplementary material available at 10.1186/s12863-022-01047-9.

## Background

Pathologic retinal neovascularization [1] is a potentially blinding consequence seen in many common diseases including diabetic retinopathy [2], retinopathy of prematurity [3], retinal vascular occlusive diseases [4], and neovascular age-related macular degeneration (AMD) [5–7] among others. Epithelial membrane protein 2 (*EMP2*) has been shown to reasonably modulate activity through neovascularization network in the retinal pigment epithelial cell line ARPE-19 [8, 9] which is reported to be important factors in progress of DR. To date, several studies have applied this approach to compare transcriptomes in vascular proliferation process within various cell types in humans [10, 11], mice [12]. However, to the best of our knowledge, there has been no genome-wide gene expression profiling study specifically comparing the transcriptom within *EMP2*-treated hRPECs.

Gene expression profiling by RNA sequencing (RNA-Seq) provides an opportunity to better understand the underlying mechanisms of vascular proliferation. Therefore, given the large number of genes potentially involved, it has been challenging to identify which are the core set of genes that play major roles in the pathways or networks. It becomes even more challenging if the recent hypothesis of omnigenic model is true for DR. Thus, it is imperative to identify disease-relevant core gene networks, and possibly the network master regulators (MRs), which, if exist, are more likely to be targeted for therapeutic interventions [13, 14]. The term MR were defined for transcription factors (TFs) and genes inflicting regulatory effects on their targets [15].

ARACNe is an unbiased algorithm that infers direct transcriptional interactions based on the mutual information between each transcriptional regulator and its potential targets [16–18]. Transcriptional regulatory networks (TRNs) conducted by ARACNe summarize the connections between transcription factors and the genes that they regulate, known as their “target genes” [19], in which case, almost all of the genes expressed in a disease-relevant cell type may confer risk for the disease through widespread network interactions with a core set of master regulators (MRs) [9]. With the constructed regulatory networks, MRs could be identified by computing the activity of each regulator (i.e., TFs, TGs, and DEGs) with the R package of VIPER (GitHub commit 0170c27).

Hence, in this study we compared *EMP2-*treated *hRPECs* and health control *hRPECs* at the transcriptome level using RNA-Seq analysis and core gene networks construction technology to identify the determinant MRs as well as the potential underlying pathyway which regulatoring vascular proliferation in *hRPECs* (Figure S1).

## Results

**Fig. 1:**
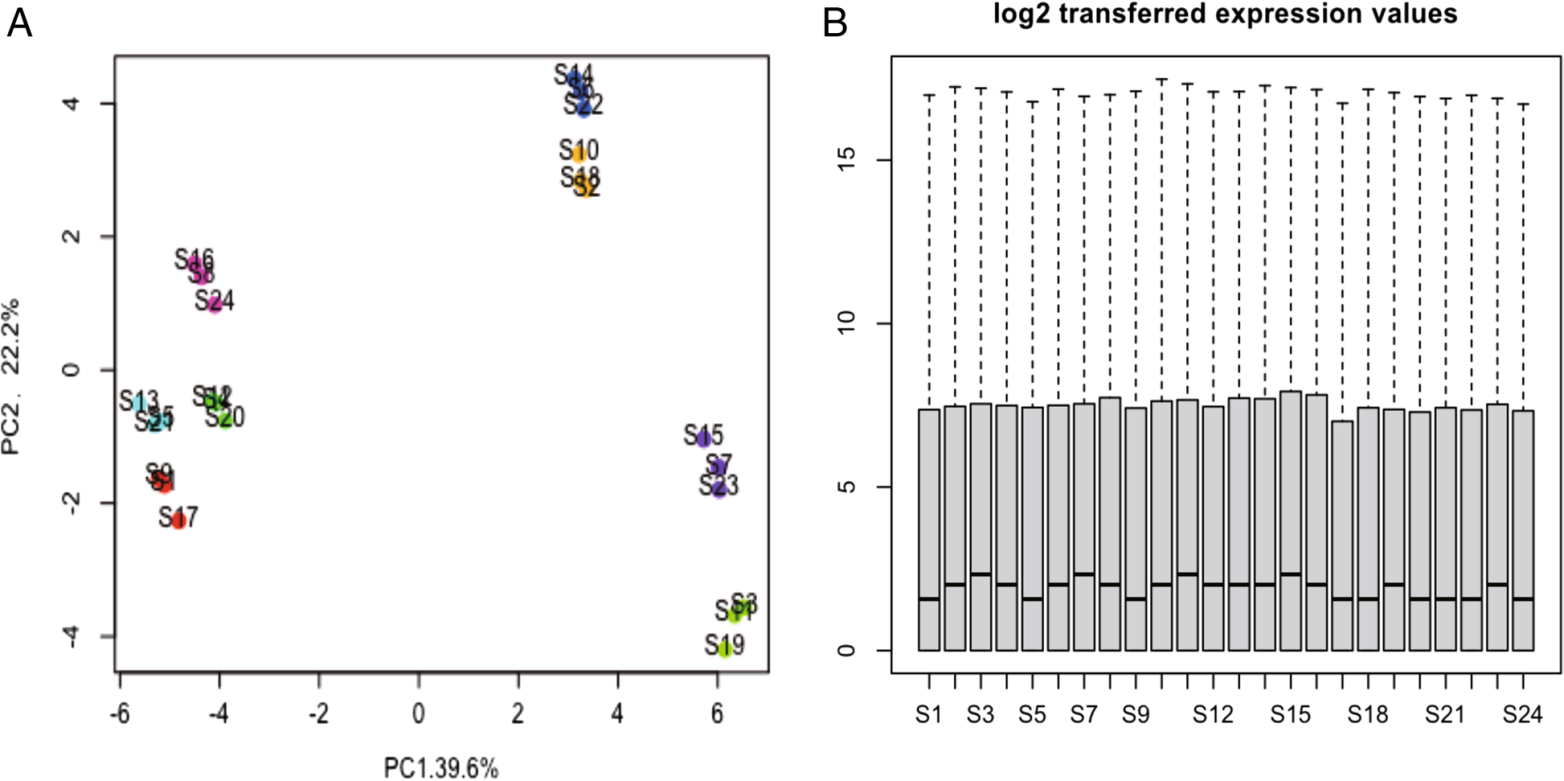
PCA and box plot of all samples. PCA scatter plot and box plot of all 24 RNA-seq samples, which were obtained based on Hisat2. PCA and box plot graphics illustrate variance of gene expression data of each sample. The percentages on each axis represent the percentages of variation explained by the principal components. Cutoff value was absolute log2FC > 1 and *P* value < 0.05. **A** PCA plot of RNA-Seq mapped reads of each sample. **B** Box plot of the log2 normalized expression values. PCA, Principal Component Analysis

All samples had high-quality RNA-seq reads with an average of 96% reads per sample. Reads with mapping quality score (MAPQ) < 10 or those with > 5 mismatches in 100 bp aligned region were discarded (Fig. 1A-B).

### Differential Expression of DEGs and functional enrichment analysis of *EMP2*-treated comparisons

**Fig. 2:**
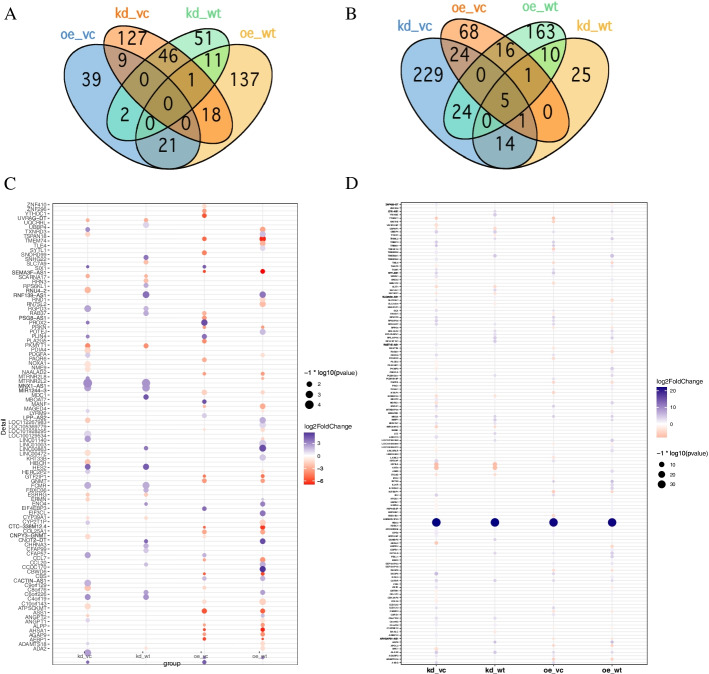
Identification of differential expressed genes (DEGs) in EMP2-regulated RPE cells. Differentially Expressed Genes (DEGs) Enrichment Venn Diagram and its bar graph. Venn diagrams were created using the web-based tool http://bioinformatics.psb.ugent.be/webtools/Venn/. Identifying DEGs and up- and down- regulated DEGs terms in deferentially expressed sequences and regulated situation in treated groups vs control groups. DESeq2 R package was used in different expression gene counting. Cutoff value was absolute log2FC > 1 and *P* value < 0.05. **A** DEGs in four comparisons under hypoxic condition, **B** DEGs in four comparisons under normoxic condition, **C-D** Up- and down- regulated DEGs in comparisons under hypoxic and normoxic condition, respectively. Colors display the expression levels of each DEGs, as pink and purple indicate down- and up-regulated genes in treated group comparing with control group, respectively. DEGs: differentially expressed genes, kd_vc: *EMP2-*knocked down *hRPECs* vs vector control *hRPECs*, kd_wt: *EMP2-*knocked-down *hRPECs* vs wild type *hRPECs*, oe_vc: *EMP2-*over expressed *hRPECs* vs vector control *hRPECs*, oe_wt: *EMP2-*over expressed *hRPECs* vs wild type *hRPECs*

**Fig. 3:**
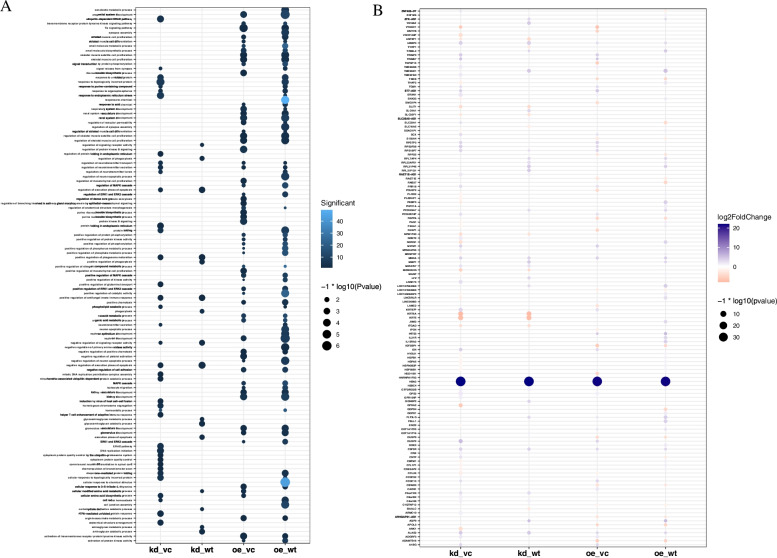
DEGs GO analysis in *EMP2*-regulated RPE cells**.** Bar graph of Gene Ontology (GO) enrichment analysis of DEGs. GO enrichment analysis terms in significant DEGs for treated group vs control. (*p* < 0.01, FDR q < 0.05, overlap cutoff > 0.5) (A) DEGs Go analysis under hypoxic condition, (B) DEGs Go analysis under normoxic condition

**Fig. 4:**
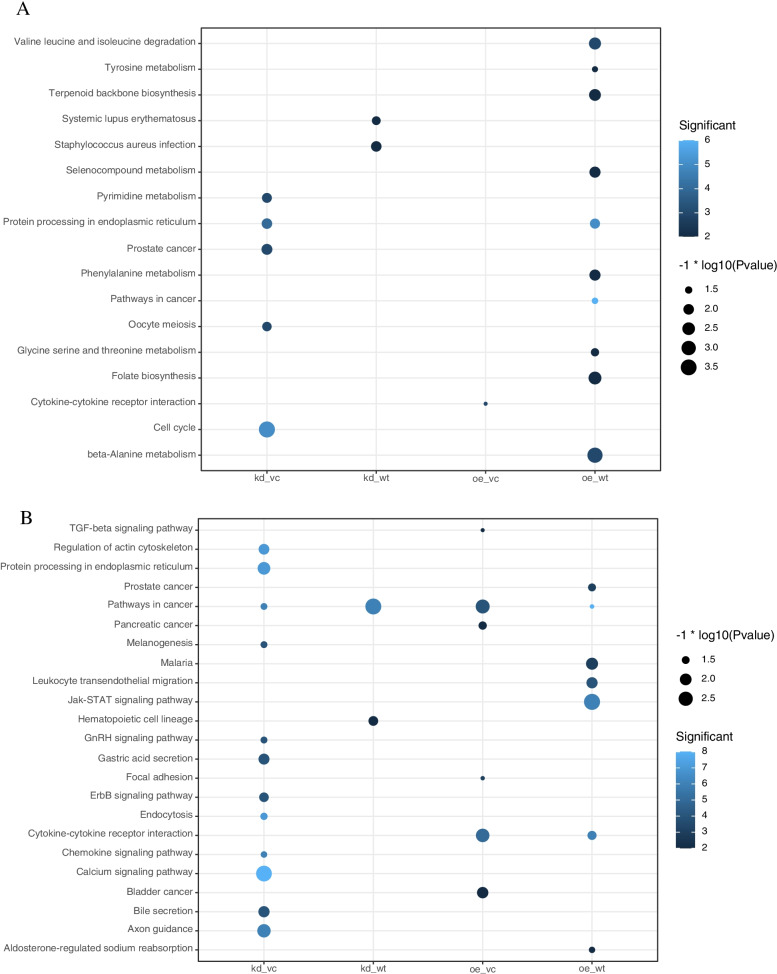
KEGG pathway analysis of the DEGs. Bar graph of Kyoto Encyclopedia of Genes, Genomes (KEGG) Pathway analysis. Enrichment of KEGG pathway terms in differentially expressed sequences for *EMP2-*treated group vs control. The RichR (https://github.com/hurlab/RichR) package was used in enrichment analysis. (*p* < 0.01, FDR q < 0.05, overlap cutoff > 0.5) (**A**) KEGG Pathway analysis of DEGs under hypoxic condition, (**B**) KEGG Pathway analysis of DEGs under normoxic condition

After low-level processing of 24 *EMP2*-treated samples and control samples (Fig. 1A-B), we performed differential expression analysis for each treated group comparing with control samples. According to the PCA plot (Fig. 1A), we compared *EMP2*-OE *hRPECs* with VC and *EMP2*-KD *hRPECs* with WT group. Totally we identified 1239 DEGs in all comparisons of *EMP2-*treated *hRPECs* versus VC or WT (Table S1). Figure 2A and B were Venn diagram of DEGs in comparisons under hypoxia and normoxia, respectively (Fig. 2A-B). Figure 2C-D showed up regulated or down regulated DEGs in comparisons under hypoxia and normoxia, respectively (Fig. 2C-D). Then we performed functional enrichment analysis and found that DEGs of *EMP2*-OE *hRPECs* vs VC under hypoxia commonly enriched in positive regulation of cell proliferation, vasculature development, protein kinase B signaling, and negative regulation of platelet activation, and enriched in metabolism and folate biosynthesis pathway(Figure S2A-S2B), which did not show in either *EMP2*-OE *hRPECs* vs VC under normoxia, or in *EMP2*-KD *hRPECs* vs WT under hypoxia or normoxia (Figs. 3 and 4).

### Transcriptome regulatory network (TRN) construction of comparisons of EMP2 treated hRPECs with control

ARACNe was run independently on the four comparisons datasets of DEGs using a conservative mutual information threshold (*p* ≤ 1.0 × 10–9, i.e., *p* ≤ 0.05 Bonferroni corrected for all candidate interactions). This resulted in highly robust TRNs of transcriptional factors (TFs) and potential target genes (TGs). To further elucidate the regulation and interaction relationship, TRNs were visualized using Cytoscape 3.7.1. In TRNs, a node is either a TG or a TF, an edge is a TF-TF, TF-TG or TG-TG relationship. Totally, 199,093 interactions were counted between 974 TFs and their inferred TGs among four comparisons (Fig. 6A-B, Tables S3-S6).

### Regulator activity inference and master regulators (MRs) identification

**Fig. 5:**
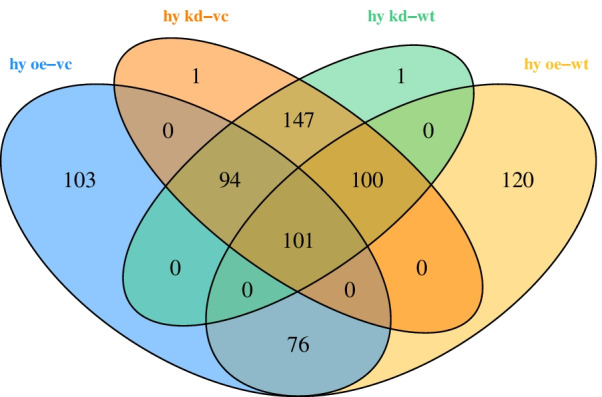
Activity of regulators of 4 comparisons under hypoxia. Venn diagram of different activity of regulators in four comparisons under hypoxia. Venn diagrams were created using the web-based tool http://bioinformatics.psb.ugent.be/webtools/Venn/. Differently expressed activity of regulators in four comparisons under hypoxia were identified. (*p* < 0.01, FDR q < 0.05, overlap cutoff > 0.5). hy kd_vc: *EMP2-*knocked down *hRPECs* vs vector control *hRPECs* under hypoxia, hy kd_wt: *EMP2-*knock-down *hRPECs* vs wild type *hRPECs* under hypoxia, hy oe_vc: *EMP2-*over expressed *hRPECs* vs vector control *hRPECs* under hypoxia, hy oe_wt: *EMP2-*over expressed *hRPECs* vs wild type *hRPECs* under hypoxia

**Fig. 6:**
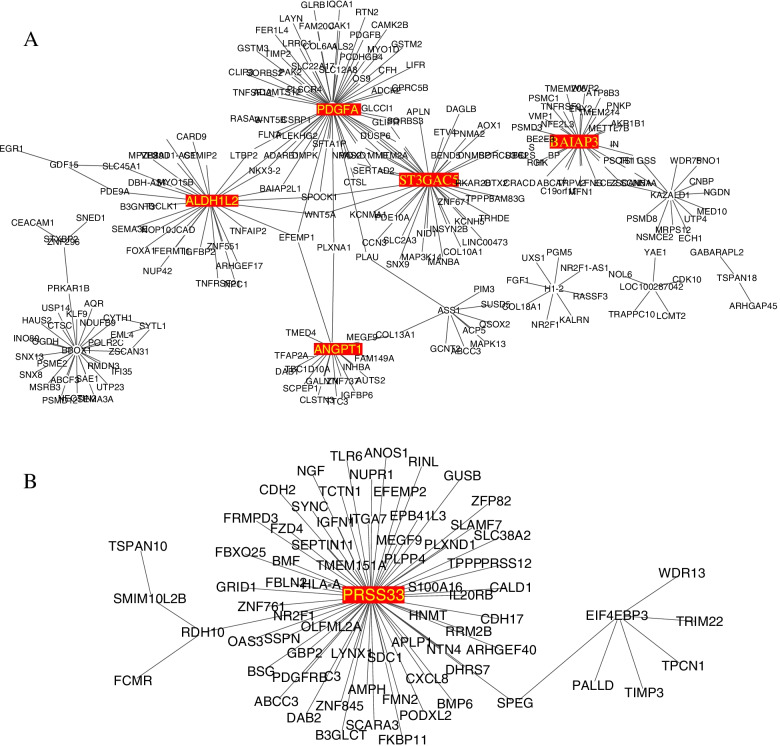
Transcription regulator networks (TRNs) of activity of regulators and DEGs. TRNs diagram of regulator activities and DEGs based on ARACNe and VIPER algorithm. TRNs diagram illustrated transcriptional factors (TFs) and potential target genes (TGs). VIPER was conducted to identify activity of TFs and TGs, then inferred potential master regulators (MRs) and mechanisms by regulating of *EMP2* in different conditions. Cytoscape software (version: 3.8.0) was used for visualizing TRNs. In this study, we defined “node” as either a TF or a TG, “edge” as a TF-TF, TF-TG or TG-TG relationship. “MR” was defined as a TF or a TG having many connections with others, which were marked with red color in the diagram. (*p* < 0.01, FDR q < 0.05, overlap cutoff > 0). **A** TRN of comparisons of *EMP2*-OE *hRPECs* vs VC under hypoxic condition, **B** TRN of comparisons of *EMP2*-KD vs WT under hypoxic condition. TRNs, transcription regulatory networks, MRs, master regulators

To further analyze the activity of identified regulators (i.e., DEGs, TFs and TGs), then inferred top MRs, the R package of VIPER was used to compute the activity of each regulator with the constructed regulatory networks. Of these, 1395 regulators were identified as high activity and their differential expression among four groups under hypoxia were compared by using Venn diagram (Table S2, Fig. 5). In present study, TRNs modules for comparisons of *EMP2-*treated *hRPECs versus VC or WT hRPECs* depicted MRs with high intramodular connectivity, i.e., genes most connected with all other genes within the module. Thus, we got *PDGFA, ALDH1L2, BA1AP3, ANGPT1 and ST3GAL5* as MRs in the TRN of hypoxia *EMP2*-OE vs VC *hRPECs* (Fig. 6A, Table S3), *PRSS33* as a MR in the TRN of hypoxia KD vs WT *hRPECs* (Fig. 6B, Table S4),

## Discussion

Since early 2010s, RNA-sequencing (RNAseq) has been used to profile transcriptome of retinal neovascularization disease. Differential expression genes (DEGs) analysis can straightforwardly analyze a gene expression dataset. Through DEGs analysis, a set of highly up-regulated and down-regulated genes can be identified by comparing two groups of samples (e.g., hypoxia vs. normoxia). However, gene expression is highly dynamic, and the expression quantification may depend on the techniques (e.g., different platforms of microarray, or RNAseq), making the cross-dataset comparison difficult. The activities of regulators cannot be directly measured by microarray or RNA-seq because these techniques only measure RNA expression level and do not consider gene activity changes by post-translational modifications. Fortunately, activity of regulators can be inferred by its regulons through VIPER, by taking RNA data and a context-specific gene regulatory network (interactome) as inputs [20–22].

In present study, transcriptome analysis of *EMP2-*over-expressed and knocked down *hRPECs* under hypoxia or normoxia elucidated eight sets of DEGs which were then used to construct TRNs along with variant analysis. TRNs analyses attempted to simplify the interactions of activity of regulators and emphasized their role in *EMP2-*treated RPE cells, then illustrated top MRs. MRs are often obscured in standard differential expression analyses (such as those including environmental stressors) and indeed many of MRs identified in our network analysis were not significant in the DEGs analysis, highlighting the strength of the network approach (Fig. 5).

Platelet derived growth factor (*PDGF)* is one of the leading vascular proliferation risk genes identified by GWAS [40]. However, the molecular mechanism underlying the genetic association remains elusive. Here, we have shown computationally that *PDGFA* is one of the top MRs of vascular proliferation factors in *hRPECs under hypoxia.* By examining the *PDGFA*-perturbed gene networks in *EMP2-* over-expressed *hRPECs,* we found that *PDGFA*-altered genes in *hRPECs* are more enriched for gene pathways that are related to proliferation and migration activities. *PDGF* families were proved to induce the proliferation and migration effects on RPE cells in proliferative retinopathy (PVR), and has been reported to participate in pericyte regulated vascular proliferation, vessel stabilization, and contribute to the formation of both the blood–brain and blood-retina barriers by regulating pericyte-endothelial cell communication [23–26]. Furthermore, we have shown that PDGF*A*-altered genes in *EMP2-*over-expressed *hRPECs under hypoxia* are more enriched for credible inflammation, oxidative stress, apoptosis GWAS risk genes [27]. Besides, we inferred that when *EMP2* was over expressed under hypoxia, *PDGFA* was upregulated and enhanced the interactions with other genes. Thus, multiple lines of evidence from our study suggest that the PDGFA gene network expression activity in the early stages of vascular proliferation may be very important for pathogenesis.

Although our computationally identification of MR has focused on *PDGFA*, other MRs that we identified here suggest that additional gene networks are relevant to vascular proliferation. The identified MRs contain many of the TFs previously reported in the literature that may potentially be involved in the vascular proliferation pathogenesis of DR, such as *ALDH1L2, BA1AP3, ANGPT1* and *ST3GAL5. ALDH1L2* (Aldehyde Dehydrogenase 1 Family Member L2) is a protein coding gene which functions as mitochondrial folate enzyme [28], the maintenance of mitochondrial integrity and energy balance of the cell [29]. The related pathways of *LDH1L2* are one carbon pool by folate and metabolism of water-soluble vitamins and cofactors (Figure S2). *ALDH1L2* has been previously proven to have correlation with *EMP2* in breast cancer cells [30]. but it was the first time to be identified in *EMP2-*treated *hRPECs*. We also noticed that *PDGFA* connected with *ALDH1L2* by *PLSCR4*. In this way, *PDGFA* may mediate accelerated ATP-independent bidirectional trans-bilayer migration of phospholipids upon binding calcium ions, and it will result in a loss of phospholipid asymmetry in the plasma membrane. This process may play a central role in the initiation of fibrin clot formation, in the activation of mast cells as well as in the recognition of apoptotic and injured cells by the reticuloendothelial system. Thus, we thought that over-expression of *EMP2* would activate *PDGFA* through *ALDH1L2-PLSCR4* pathway by initializing fibrin clot and recognizing apoptosis.

We also identified that BAI1 Associated Protein 3 (*BAIAP3*) had correlation with *MMP16* [31]. *BAIAP3*, functioning in endosome to Golgi retrograde transport, may regulate behavior and food intake by controlling calcium-stimulated exocytosis of neurotransmitters including NPY and serotonin and hormones like insulin. Such neurotransmitters have been proven to have the ability to degrade various components of the extracellular matrix, such as collagen type III and fibronectin in the pathogenesis of diabetic cataract [17].

This investigation was mainly based on ARACNe algorithm analysis of transcriptome information of *hRPECs* treated by hypoxia and exogenous *EMP2*. The TRN constructed in this paper provided clues of interaction mechanism between these genes, which not only provided theoretical support for revealing the regulatory relationship among them, but also provided candidate genes, i.e., up-and down-regulated pathway genes, transcriptional factors, and MRs, which could be studied for future SNP discovery work to establish association with different phenotype or traits of interest. We also established a scientific exploration mode for exploring effective targets of anti-neovascularization, which made the follow-up basic verification and clinical research more reliable. However, the results of bioinformatics analysis ultimately need to be verified by many experiments and combined with clinical practice to carry out gene function and mechanism verification for substantive exploration.

## Conclusions

In summary, by using a computational approach, we identified *PDGFA, ALDH1L2, BA1AP3, ANGPT1* and *ST3GAL5* as MRs that likely contributed to *EMP2*-induced vascular proliferation in *hRPECs* under hypoxia. Although powerful, we acknowledged the limitation of our approach in identifying top MRs, because selecting a top MR was not purely “data-driven”; for instance, *PDGFA ALDH1L2, BA1AP3, ANGPT1 and ST3GAL5* were identified as top MRs among many other MRs was not only data-driven but also based on prior knowledge about their association with vascular proliferation. Nonetheless, our study suggested that MRs in vascular proliferation in *EMP2* and hypoxia treated *hRPECs* could be identified by transcriptional network construction and that MRs such as *PDGFA* as well as other MRs might constitute convergent gene networks that confer disease risk in a spatial and temporal manner. Therefore, *PDGFA* and other identified MRs might be a building part of vascular proliferation architecture, which collectively drived DR onset and progression. Transcriptional network construction in larger and more DR-relevant cell types/stages, combined with empirical network perturbation, would further pave the way to deepen our understanding of the genetic contribution to the complex biology of vascular proliferation during DR disease biology.

## Methods

### Data availability

The RNA-seq data profiles were obtained from the NCBI GEO database (https://www.ncbi.nlm.nih.gov/geo/) GSE151610. The dataset contained 24 samples, which contained mRNA profiles of *hRPECs* panel consisting of *EMP2-*knocked-down (KD) *hRPECs*, *EMP2-*over-expressed (OE) *hRPECs*, vector control (VC) *hRPECs*, and wild type (WT) *hRPECs* under hypoxia or normoxia. This project was approved by the Institutional Review Board of Sir Run Run Hospital, Nanjing Medical University. All procedures of this study complied with the protocol.

### Identification of DEGs

The data set is available at the NCBI Short-Read Archive (SRA), under accession SRA265560. The RNA-Seq data set was mapped in the Homo sapiens (human) genome assembly GRCh38 (hg38) by Hisat2. Feature Counts was used in read counts. After filtering all 0 expression genes, DESeq2 R package was used in different expression gene counting. Cutoff value was absolute log2FC > 1 and *P* value < 0.05.

### GO and KEGG pathway enrichment analysis

We performed functional enrichment analysis using Kyoto Encyclopedia of Genes, Genomes (KEGG) pathway and Gene Ontology (GO) with a *p* value < 0.05 as the significance cutoff. The RichR (https://github.com/hurlab/RichR) package was used in enrichment analysis (Figure S1).

### Transcription regulatory network construction

Regulatory networks were constructed by ARACNe (Algorithm for the Reconstruction of Accurate Cellular Networks) [32–34] using significant DEGs. First, mutual interaction between a candidate TF(x) and its potential target (y) was computed by pairwise MI, MI(x, y), using a Gaussian kernel estimator. A threshold was applied on MI based on the null hypothesis of statistical independence (*P* < 0.05, Bonferroni-corrected for the number of tested pairs). Secondly, the constructed network was trimmed by removing indirect interactions using the data processing inequality (DPI), a property of the MI. Parameters was set to zero DPI (Data Processing Inequality) tolerance and MI (Mutual Information) *p*-value threshold of 10 − 8. Therefore, for each (x, y) pair, a path through another TF(z) was considered, and every path pertaining to the following constraint was removed: MI (x, y) < min (MI (x, z),MI (z, y)). A *P* value threshold of 1 × 10 − 8 using DPI = 0.1 (as recommended [35]) was used when running ARACNe. Cytoscape software (version: 3.8.0) was used for visualizing the regulatory networks.

### Regulator activity inference

The VIPER algorithm had proven to be a useful tool for estimating regulator activity from gene expression data using enriched regulon analysis. With the constructed regulatory networks, we computed the activity of each regulator (i.e., TFs, TGs, and DEGs) using the R package of VIPER (GitHub commit 0170c27). The output of VIPER is a list of highly active MRs as well as their activity scores and their enrichment *P* values. A set of regulators with significant activity difference were defined at adjusted *p*-value < 0.01.

### Statistical test

All statistical tests and plotting were performed in R 3.5.2 (https://www.r-project.org/) unless otherwise mentioned.

## Supplementary Information

**Additional file 1.**

**Additional file 2**.

## Data Availability

The datasets analyzed during the current study are available in the NCBI GEO database (https://www.ncbi.nlm.nih.gov/geo/) GSE151610.

## Abbreviations

MRs: Master regulators
*EMP2*: Epithelial membrane protein 2
hRPECs: Human retinal pigment epithelial cells
TFs: Transcription factors
TGs: Target genes
DEGs: Differential expression genes
VC: Victor control
WT: Wild type
ARACNE: Algorithm for the Reconstruction of Accurate Cellular Networks
VIPER: Virtual Inference of Protein activity by Enriched Regulon analysis
TRNs: Transcription regulatory networks
AMD: Age-related macular degeneration
OE: Over-expressed
KD: Knocked-down
SRA: Short-Read Archive
KEGG: Kyoto Encyclopedia of Genes, Genomes
GO: Gene Ontology
