# Supplementary material for: Network-based inference of master regulators in epithelial membrane protein 2-treated human RPE cells
Source: BMC Genom Data. 2022 Jul 7;23:52. doi: 10.1186/s12863-022-01047-9 (PMC9264685; doi:10.1186/s12863-022-01047-9)
Supplement: Supplementary file 2 — Additional file 2. [file 12863_2022_1047_MOESM2_ESM.docx]

Supplementary

***Figure Legends***

Figure S1 The analysis workflow of this study.

Figure S2 Pathyway of ALDH1L. (A) hsa00670, One carbon pool by folate, (B) hsa01100, Metabolic pathways.


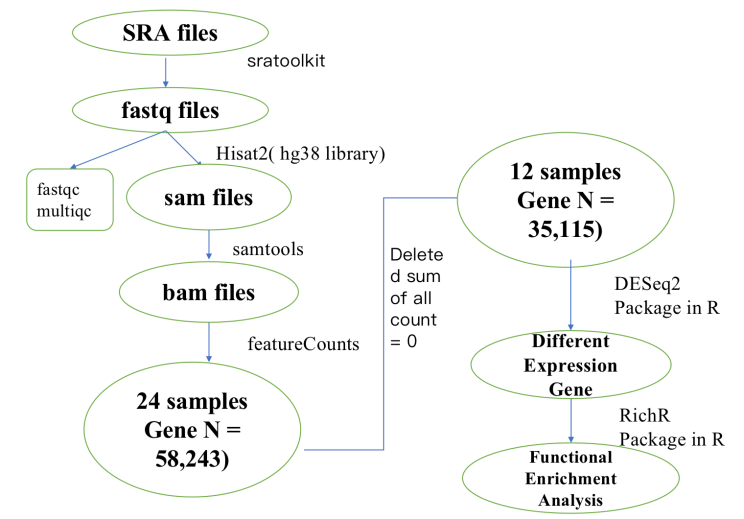


Figure S1 The analysis workflow of this study.


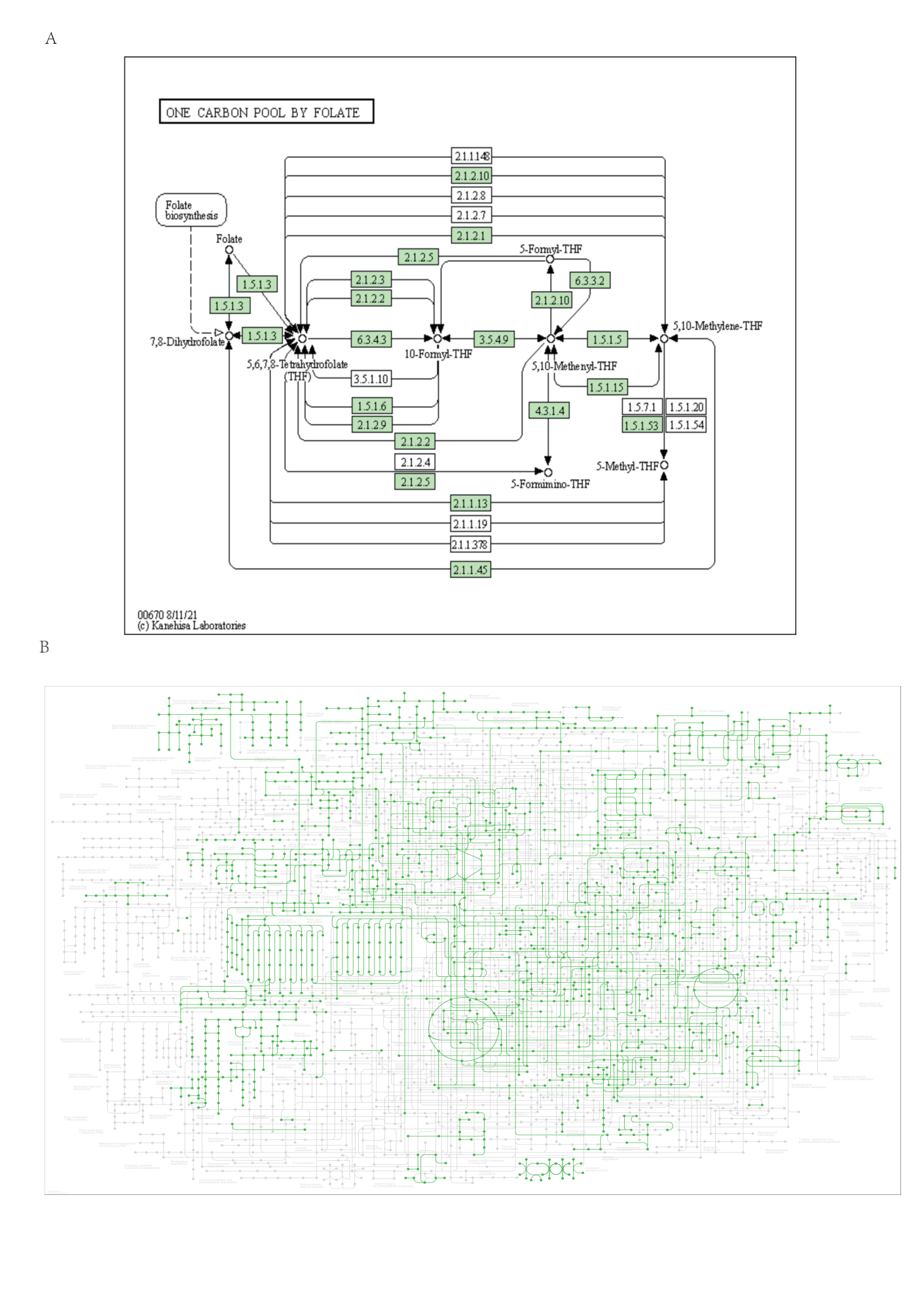


Figure S2 Pathyway of ALDH1L. (A) [hsa00670](https://www.kegg.jp/pathway/hsa00670+160428), One carbon pool by folate, (B) [hsa01100](https://www.kegg.jp/pathway/hsa01100+160428), Metabolic pathways
